# Sleeve lobectomy by video-assisted thoracic surgery versus thoracotomy for non-small cell lung cancer

**DOI:** 10.1186/s13019-015-0318-6

**Published:** 2015-09-10

**Authors:** Shijie Zhou, Guotian Pei, Yi Han, Daping Yu, Xiaoyun Song, Yunsong Li, Ning Xiao, Shuku Liu, Zhidong Liu, Shaofa Xu

**Affiliations:** Department of Thoracic Surgery, Beijing Chest Hospital, Capital Medical University, Machang 97, Tongzhou District Beijing, 101149 China

**Keywords:** VATS, thoracotomy, lung cancer surgery

## Abstract

**Background:**

Both video-assisted thoracic surgery (VATS) and thoracotomy are used for sleeve lobectomy for patients with non-small cell lung cancer (NSCLC). This retrospective study aimed to assess the safety and efficacy of VATS sleeve lobectomy for NSCLC patients.

**Methods:**

Between May 2009 and May 2013, 51 sleeve lobectomies (10 by VATS and 41 by thoracotomy) were performed for patients with NSCLC. Operative characteristics and postoperative course were compared between two groups.

**Results:**

Patient demographics were similar between the two groups. Thoracotomy patients had larger tumors compared with VATS patients (*p* = 0.02). VATS patients had a longer operating time (*p* < 0.001) but a shorter length of postoperative hospital stay (*p* = 0.009). The two groups did not differ in pathologic stage, histologic results, blood loss, ICU stay, amount of chest drainage, duration of chest drainage, numbers and distributions of dissected lymph nodes and the occurrence of complications. There were no perioperative deaths in the VATS group, whereas there was one death (2.4 %) in the thoracotomy group. There were no conversions to thoracotomy in the VATS group. The overall median survival between the two groups was similar (3.2 years VATS versus 3.2 years thoracotomy, log-rank *p* = 0.58).

**Conclusions:**

VATS sleeve lobectomy for the treatment of NSCLC is technically feasible and safe and is associated with comparable complication rates and survival compared with thoracotomy approach, but it deserves further investigation in large series.

## Background

Sleeve lobectomy, which is featured by not only the maximal resection of tumors but also the maximal reservation of the normal lung tissues and lung functions, was considered as an alternative procedure to pneumonectomy for patients with central lung cancer [1–4]. Although video-assisted thoracic surgery (VATS) is regarded as a minimally invasive procedure with good long-term survival outcomes [5], many surgeons considered that sleeve resection was an absolute contraindication for VATS lobectomy due to oncological concerns in obtaining a complete resection and technical complexity. Therefore, there are few reports in the literature of VATS sleeve lobectomy for lung cancer in recent years [6–14]. In addition, the purported benefits of VATS over thoracotomy in performing sleeve lobectomy have only been reported in series using only one surgical approach or in single case reports.

In this study, we conducted a retrospective study to examine the safety and efficacy of the video-assisted technique in the performance of sleeve lobectomies for NSCLC patients and to compare the outcome from these procedures with that from sleeve lobectomies performed through a standard thoracotomy.

## Methods

### Patients

**Fig. 1:**
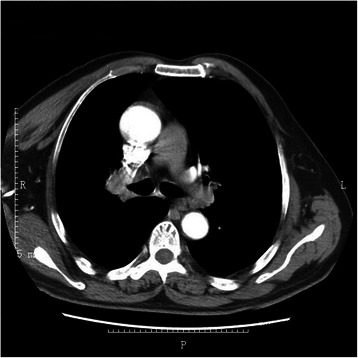
Chest computed tomography showing a tumor located around bronchus of the right upper lobe

**Fig. 2:**
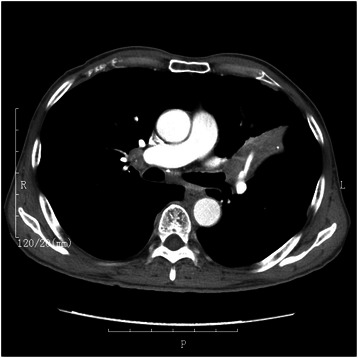
Chest computed tomography showing a tumor located around bronchus of the left upper lobe

We retrospectively reviewed the files of 10 patients who underwent a sleeve lobectomy for non-small cell lung cancer (NSCLC) by the VATS approach in the Department of Thoracic Surgery at Beijing Chest Hospital, Capital Medical University, between May 2009 and May 2013. The inclusion criteria for VATS sleeve lobectomy include: endobronchial tumors and small tumors (tumor size <5cm) with limited invasion of the bronchus; no evidence of vessel invasion; no direct invasion to the surrounding organs; no extensive pleural adhesion on the CT scan;. and the ability to physiologically tolerate the planned resection (Fig. 1). To better evaluate the safety and effectiveness of the VATS approach, we reviewed the files of 41 patients who underwent a sleeve lobectomy for NSCLC by the open approach during the same period at the same hospital (Fig. 2). Preoperative workup included physical examination, chest roentgenography, computed tomography of the chest and upper abdomen, computed tomography or magnetic resonance imaging of the brain, pulmonary function assessment, arterial blood gases, bronchofiberscopy, electrocardiography, and bone scintigraphy. Mediastinoscopy and positron emission tomography were performed if necessary. The study was approved by the ethics committee of Beijing Chest Hospital, Capital Medical University, and all enrolled patients provided written informed consent.

### Data collection

Patient demographics, preoperative investigations, tumor characteristics, intraoperative details, and postoperative course were recorded. Histologictyping occurred according to The World Health Organization Histologic Typing of Lung Tumors. The postsurgical (pathologic) stages of the patients were based on the seventh TNM Classification of Malignant Tumors [15]. Postoperative complications were defined as those occurring within 30 days of surgery. Hospital mortality included all deaths during the first 30 days after operation, or during the postoperative hospital stay.

### Surgical technique

All operations were performed by two surgeons (Z.L. and S.X.) with extensive experience in thoracoscopic and open procedures from the same department. The decision to employ either a VATS or thoracotomy approach was made by the surgeons. All patients underwent standard anesthesia care with the use of double-lumen endotracheal tubes and perioperative fluid restriction. All patients were performed in the lateral decubitus position.

**Fig. 3:**
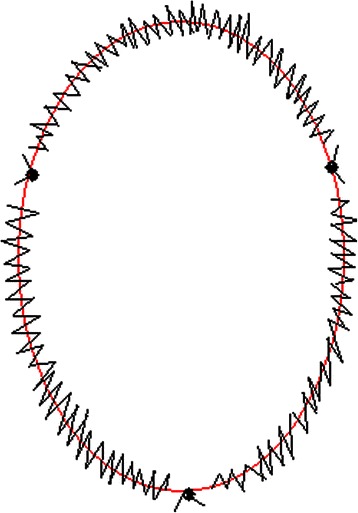
Bronchial anastomosis was performed using continuous suture and three points interrupted suture

The main difference in the conduct of the operation between VATS lobectomy and VATS sleeve lobectomy is the need for a bronchial anastomosis. For VATS patients, we choose the same incisions as the VATS lobectomy: a 10-mm camera port placed in the eighth intercostal space (ICS) at the midaxillary line; a 4-cm to 5-cm anterior utility incision in the fourth or fifth ICS; a 10-mm incision in the auscultatory triangle. Endoshears/Endokittners (U.S. Surgical, Norwalk, CT, USA) were used for dissection. Pulmonary vessels were resected as for standard lobectomy using an EndoGIA vascular stapler (U.S. Surgical, Norwalk, CT, USA).The bronchus was divided using scissors. The bronchial stump was then assessed by frozen sectioning to be pathologically free of cancer. After pathologic examination, end-to-end bronchial anastomosis was performed. In the first five cases, the bronchial anastomosis was performed using interrupted sutures of 3-0 polyglactin 910 (Vicryl®, Ethicon Inc., Somerville, NJ, USA). Interrupted sutures were first placed on the cartilaginous portions of the bronchial orifices, then on the membranous portions. All sutures were held together in the pleural cavity by agrasper inserted through the other access port. After placing all sutures, knot tying was started with a forceps-type tying instrument at the suture placed at the deepest position. All sutures were tied, and then the bronchoplasty was finally completed. In the later five cases, continuous suture and three points interrupted suture were performed using Vicryl® (Ethicon Inc., Somerville, NJ, USA) 3-0 sutures for saving anastomosis time (Fig. 3). The anastomosis was first initiated at the junction of the cartilaginous and membranous walls by using interrupted 3-0 polyglactin 910 (Vicryl®, Ethicon Inc., Somerville, NJ, USA) suture. Absorbable 3-0 sutures, with knots placed outside the bronchial lumen, are used to decrease the possibility of stricture and granuloma formation. Then followed by the continuous suturing clockwise and counter clockwise for one third of the circumference. Another two points of interrupted suturing is followed when thecontinuous suturing is finished. The final step is to complete the remaining one third of the bronchus circumference using continuous suture. A complete systemic mediastinal lymph node dissection was performed after bronchial reconstruction. The resected lobe was placed in an Endocatch bag (U.S. Surgical, Norwalk, CT, USA). A sealing test was performed to confirm that there was no leakage after completion of the anastomosis. Two 28F chest tubes were placed and the incisions were closed. Postoperative bronchoscopy was performed to clear blood and secretions, and to confirm that there was no stenosis after the operation.

For thoracotomy patients, sleeve lobectomy was performed though a traditional posterolateral thoracotomy incision at the fifth intercostal space. The surgical technique used in the thoracotomy approach was similar to that of VATS, except for the method of the bronchial anastomosis, which was performed using several interrupted3-0 absorbable sutures in all thoracotomy patients.

### Postoperative management

All patients were extubated in the operating or recovery room and were kept in the intensive care unit (ICU) until stable. All patients had intraoperative and postoperative antibiotic prophylaxis. Patient controlled intravenous analgesia (PCIA) for postoperative pain relief was offered to all patients regardless of the planned operative approach. The chest tube was removed if the drainage volume was less than 200 ml per day and no air leakage was observed. Fiberoptic bronchoscopy was routinely performed before discharge.

### Statistical analysis

The data are presented as frequency and percentage for categoric variables and as mean ± standard deviations for continuous variables. The Wilcoxon rank test were used to compare continuous variables and the χ^2^ or Fisher exact tests were used for categorical variables. Survival was recorded from the day of surgery until death or the last follow-up contact. Survival analysis was estimated by the Kaplan-Meier method and compared by the log-rank test. A *p* value less than 0.05 was considered to be significant. The statistical software SPSS 16.0 (SPSS Inc., Chicago, IL, USA) was used for all analyses.

## Results

During the study period, 51 patients, 10 VATS (20 %) and 41 thoracotomy (80 %), underwent sleeve lobectomy for NSCLC. Demographics, baseline characteristics, and comorbidities of the VATS and thoracotomy patients are shown in Table 1. There were more male patients in both groups. Comorbidities and preoperative pulmonary function values were comparable between two groups. No patient had a history of neoadjuvant treatments or thoracotomy in both two groups.

**Table 1:** Patient characteristics

Variable	Thoracotomy (n = 41)	VATS (n = 10)	*p* Value
Gender	35M/6F	9M/1F	1.00
Age (y)	62.5 ± 7.2	60.5 ± 16.9	0.72
Tobacco use	16 (39.0 %)	3 (30.0 %)	0.61
Comorbidities^a^			
Hypertension	10 (24.4 %)	4 (40.0 %)	0.33
Diabetes mellitus	3 (7.3 %)	1 (10.0 %)	0.78
Coronary artery disease	8 (19.5 %)	2 (20.0 %)	0.97
COPD	8 (19.5 %)	2 (20.0 %)	0.97
Pulmonary function			
FVC (L)	3.1 ± 0.6	3.2 ± 0.3	0.38
FEV1 (L)	2.2 ± 0.5	2.3 ± 0.2	0.11
FEV1/FVC %	70.3 ± 8.9	73.5 ± 4.8	0.14

^a^Some patients had one or more comorbidities

COPD = chronic obstructive pulmonary disease; FEV1 = forced expiratory volume in 1 second; FVC = forced vital capacity

Tumor characteristics and operative details are listed in Table 2. Thoracotomy patients had larger tumors compared with VATS patients (*p* = 0.02). Right upper sleeve lobectomy was the most common procedure in both two groups. No statistically significant difference was found in either the distribution of pathologic stages or the histologic results between two groups. The most common histologic type in both two groups was squamous cell carcinoma. The mean number of lymph nodes resected and the distribution of resected nodal status were similar between two groups. The average operating time was significantly longer in the VATS group than in the thoracotomy group (226 ± 37 min versus166 ± 40 min, *p* < 0.001). There were no conversions to thoracotomy in the VATS group. As shown in Table 2, no statistically significant difference was found between two groups in terms of the estimated blood loss, amount of chest drainage, duration of chest drainage, and the length of intensive care unit(ICU) stay. However, the mean length of postoperative hospital stay was significantly shorter in the VATS group than in the thoracotomy group (11.6 ± 2.8days vs. 16.1 ± 4.9 days, *p* = 0.007).

**Table 2:** Tumor characteristics and operative details

Variable	Thoracotomy	VATS	*p* Value
Tumor size (cm)	3.6 ± 1.2	2.7 ± 0.9	0.02
Total lymph nodes	22.0 ± 8.3	25.7 ± 6.5	0.20
Total lymph node stations	6.8 ± 1.4	6.4 ± 0.7	0.43
Histologic Type			0.55
Squamous cell carcinoma	33 (80.5 %)	8 (80.0 %)	
Adenocarcinoma	7 (17.1 %)	1 (10.0 %)	
Other^a^	1 (2.4 %)	1 (10.0 %)	
Pathologic stage			0.36
I	18 (43.9 %)	6 (60.0 %)	
II	10 (24.4 %)	2 (20.0 %)	
IIIa	13 (31.7 %)	2 (20.0 %)	
Type of lung resection			0.31
Right side	30 (73.2 %)	7 (70.0 %)	
Left side	11 (26.8 %)	3 (30.0 %)	
Upper lobe	24 (58.5 %)	6 (60.0 %)	
Middle lobe	1 (2.4 %)	1 (10.0 %)	
Lower lobe	8 (19.5 %)	3 (30.0 %)	
Upper and middle lobe	2 (4.9 %)	0	
Lower and middle lobe	6 (14.6 %)	0	
Operating time (min)	166 ± 40	226 ± 37	<0.001
Estimated blood loss (mL)	318 ± 198	406 ± 200	0.22
Intensive care unit (hours)	20.3 ± 1.7	19.1 ± 1.6	0.06
Chest tube drainage (mL)	2229 ± 1508	2247 ± 990	0.97
Chest tube duration (days)	8.6 ± 5.1	7.5 ± 1.7	0.50
Postoperative hospital stay (days)	16.1 ± 4.9	11.6 ± 2.8	0.009

^a^Other results included carcinoid, or neuroendocrine carcinoma

The various postoperative complications are listed in Table 3. Onepatient (10.0 %) of the VATS group and 10 (24.4 %) of the thoracotomy group experienced postoperative complications (*p* = 0.62). In addition, one patient in the thoracotomy group experienced bronchopleural fistula on the 46th postoperative day. The patient was treated with bronchoscopic interventions using glues and discharged two weeks later. There were no perioperative deaths in the VATS group, whereas there was one death (2.4 %) in the thoracotomy group although this difference was not statistically significant. This patient died of pulmonary embolus 15 days after operation.

**Table 3:** Complications

Complication	Thoracotomy	VATS	*p* Value
Prolonged air leak	2	0	
Atrial fibrillation	1	1	
Atelectasis	2	0	
Pneumonia	3	0	
Chylothorax	1	0	
Bronchopleral fistula^a^	0	0	
Pulmonary embolus	1	0	
Total	10 (24.4 %)	1 (10.0 %)	0.57

^a^One patient in the thoracotomy group experienced bronchopleural fistula on the 46th postoperative day

**Fig. 4:**
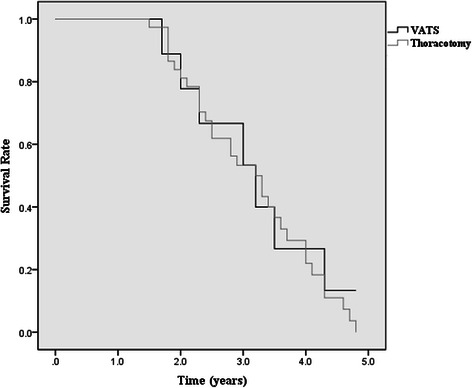
Kaplan-Meier curve for overall survival by VATS (black line) and thoracotomy (gray line)

All patients completed follow-up and all patents (*n* = 51) were included in the survival analysis. Median follow-up was 34 months in both groups. The overall median survival between the two groups was similar (log-rank, *p* = 0.58) (Fig. 4). For the VATS group, the median survival estimate was 3.2 years; the overall 1-, 2-, 3-, and 4-years survival rates were 100 %, 89 %, 73 %, and 40 % respectively. For the thoracotomy group, the median survival estimate was 3.2 years; the overall 1-, 2-, 3-, and 4-years survival rates were 100 %, 84 %, 63 %, and 56 % respectively.

## Discussion

Since the technique of VATS lobectomy was first described in the early 1990s, a number of studies have demonstrated its safety and advantages [16–18]. During the following years, VATS lobectomy gained widespread acceptance in the treatment of lung cancer, and eventually written into the guideline for the diagnosis and treatment of lung cancer [19]. However, the application of VATS sleeve lobectomy has been somewhat limited due to oncological concerns in obtaining a complete resection and technical complexity. Since the first case of VATS sleeve lobectomy was reported in 2002 [20], more and more studies had described the application of VATS sleeve lobectomy include uniportal VATS bronchial sleeve lobectomy [9–11] and even thoracoscopic double sleeve lobectomy [12, 13]. In this study we reviewed our experience with open and thoracoscopic sleeve lobectomy in order to prove the safety and efficacy of the VATS procedure.

The results of the current study demonstrate that VATS sleeve lobectomy can be safely performed with a favorable postoperative outcome compared with the thoracotomy approach. The VATS approach provides an obvious advantage of the thoracotomy technique in the aspect of postoperative hospital stay. However, the VATS group had a longer operating time than the thoracotomy group. It is because VATS patients require a significant longer anastomosis time than thoracotomy patients. The average anastomosis time in the VATS group, which recorded using a prospective database, was 55 ± 18 min. Like all other procedures, VATS sleeve lobectomy also has a learning curve for both the surgeon and the assistant. It is believed that the perioperative outcomes such as operating time and anastomosis time would be reduced after the initial learning period.

Due to oncological concerns of complete resection and technical complexity, only small tumors with limited invasion of the main bronchus without major invasion of the pulmonary artery were selected for VATS sleeve lobectomy by careful preoperative computed tomography of the chest and bronchofiberscopy. In the present study, there were no conversions to thoracotomy in the VATS group. The same results have been reported in several previous studies [6, 7, 21, 22]. Interference by lymph nodes and bleeding are the most important causes of conversion to thoracotomy in video-assisted thoracoscopic surgery. Patient selection bias may have played a role in the favorable results. Indications for VATS sleeve lobectomy as well as thresholds for conversions vary among surgeons, and these factors change over time as the surgeon gains more experience with the procedure.

The major complications after sleeve lobectomy include pneumonia, secondary atelectasis, and bronchial complications [23, 24]. However, bronchial anastomotic complications including bronchovascular and bronchopleral fistula, anastomotic stricture, and anastomotic dehiscence are probably the most serious complications associated with sleeve lobectomy. In our present study, only one patient in the thoracotomy group experienced bronchopleural fistula on the 46th postoperative day, whereas there was no bronchial anastomotic complications in the VATS group. The postoperative complication rates after VATS sleeve lobectomy vary in different reports. Mahtabifard et al. reported that four of 13 (31 %) patients undergoing VATS sleeve lobectomy experienced postoperative complications, including atrial fibrillation, anastomotic stricture, reintubation, and bronchial tear requiring repair [7]. Wang et al. have described the successful use of VATS sleeve lobectomy with only one patient in their cohort of 15 (6.7 %) experiencing minor complications [22]. Similarly, Agasthian reported that only one patient (4.8 %) developed bronchopleural fistula in a cohort of 21 patients who underwent VATS bronchoplasy [8]. Conversely, some studies have indicated that VATS sleeve lobectomy can be performed safely without major postoperative complications [6, 21]. Such variability probably depends on the characteristics of studied populations, the surgeon’s skill-level, and postoperative management. In our study, although there was only one patient (10 %) experienced atrial fibrillation and no death occurred in the VATS group, no significant differences were found between the two groups in terms of morbidity and mortality. Also, the incidence of postoperative complications and mortality rate in our series were in agreement and compare favorably with previous reports [8, 22]. Patient selection bias and low statistical power may account for this observation.

Our findings support those of previously published reports that have demonstrated that complete mediastinal lymph node dissection through a VATS approach is feasible and that results are equivalent to those of a thoracotomy approach. Sugi et al. found no difference between the numbers of lymph nodes dissected among VATS versus open group during lobectomy [25]. Similarly, Watanabe et al. conducted a retrospective review of 770 patients with cN0-pN2 NSCLC (VATS = 450, open = 320) and found no difference in terms of the total number of lymph nodes, number of lymph node stations, number of mediastinal nodes and mediastinal stations between VATS and open lobectomy [26]. Data from the recent ACSOG Z0030 trial also showed no difference between VATS and thoracotomy for node dissection [27].

The best measure of any cancer treatment is survival. To date, no studies have reported the long-term outcomes after VATS sleeve lobectomy. In our study, although we could not compare the 5-year survival rate after sleeve lobectomy between VATS and thoracotomy due to the short-term follow up, the median survival between the two groups was similar and no significant differences were found between the two groups in 1-, 2-, 3-, and 4-years survival rates. It appears that a VATS approach does not compromise the survival for lung cancer patients.

A full-thickness interrupted suture is the most popular technique for anastomosis and is widely employed. Mckenna et al. performed interrupted suturing with 3-0 Vicryl sutures [7]. Of the 13 patients reported, two (15.4 %) had bronchial anastomotic complications including anastomotic stricture and bronchial tear requiring repair. However, the end-to-end anastomosis could also be performed by complete continuous suture. The safety of the continuous suturing technique has been reported by Kutlu et al. in a series of 100 patients using 3-0 propylene sutures with 2 % bronchopleural fistula, 1 % bronchovascular fistula, and 5 % late stenosis [28]. The other technique involves closing the membranous portion of the bronchus with a simple continuous suture, and the closure of the cartilaginous portion with simple interrupted sutures [29]. Although interrupted anastomosis is relatively reliable, the sutures were sometimes placed with the knots in the bronchial lumen, which usually resulted in postoperative sputum retention, irritable cough, and anastomotic stricture. In contrast, continuous anastomosis avoided entanglement of the sutures and prevented against too much tightening of the anastomotic sites. However, the limitation of the continuous anastomosis is that one-site interruption of the suture would lead to anastomosis failure. Continuous anastomosis combined with interrupted consolidation used in our study avoided the presence of absorbable suture placing in the bronchial lumen, therefore decreasing postoperative sputum retention and irritable cough. It also decreased the anastomosis time. In addition, some authors believe that bronchial anastomotic complications can be prevented by precise dissection, preservation of bronchial blood supply, and interposition of pedicled tissue between the bronchial and vascular structures [30].

Some limitations in the present study need to be acknowledged. First, the main limitation of this study is the non-randomized and retrospective nature. Unknown confounding variables and imbalance between patient characteristics, such as tumor size, could bias the results. Second, VATS sleeve lobectomy was performed for only 10 patients during our study period due to its technical complexity. The small sample size can have effects on the outcome and worth of the study. Third, although the current data have shown that VATS sleeve lobectomy can produce similar outcomes with open surgery, it must be borne in mind that the selection bias for VATS sleeve lobectomy may predispose them to an improve outcome.

## Conclusions

Despite a small sample size, we can preliminarily conclude that VATS sleeve lobectomy is technically feasible and safe for selected patients in specialized centers. VATS sleeve lobectomy can be performed with comparable complication rates and survival compared with the thoracotomy approach. However, VATS sleeve lobectomy is associated with a significantly decreased length of hospital stay. The non-randomized retrospective design might make multiple factors have biased the results. Therefore, further randomized controlled studies with large series may be necessary.

## Abbreviations

NSCLC: non-small cell lung cancer
VATS: video-assisted thoracic surgery
ICU: intensive care unit
PCIA: patient controlled intravenous analgesia

## References

[1] Gaissert HA, Mathisen DJ, Moncure AC, Hilgenberg AD, Grillo HC (1996). Survival and function after sleeve lobectomy for lung cancer. J Thorac Cardiovasc Surg..

[2] Okada M, Yamagishi H, Satake S, Matsuoka H, Miyamoto Y (2000). Survival related to lymph node involvement in lung cancer after sleeve lobectomy compared with pneumonectomy. J Thorac Cardiovasc Surg..

[3] Ferguson MK, Lehman AG (2003). Sleeve lobectomy or pneumonectomy: optimal management strategy using decision analysis techniques. Ann Thorac Surg..

[4] Deslauriers J, Gregoire J, Jacques LF, Piraux M, Guojin L (2004). Sleeve lobectomy versus pneumonectomy for lung cancer: a comparative analysis of survival and sites or recurrences. Ann Thorac Surg..

[5] Cao C, Zhu ZH, Yan TD, Wang Q, Jiang G (2013). Video-assisted thoracic surgery versus open thoracotomy for non-small-cell lung cancer: a propensity score analysis based on a multi-institutional registry. Eur J Cardiothorac Surg..

[6] Nakanishi K (2007). Video-assisted thoracic surgery lobectomy with bronchoplasty for lung cancer: initial experience and techniques. Ann Thorac Surg..

[7] Mahtabifard A, Fuller CB, McKenna RJ (2008). Video-assisted thoracic surgery sleeve lobectomy: a case series. Ann Thorac Surg..

[8] Agasthian T (2013). Initial experience with video-assisted thoracoscopic bronchoplasty. Eur J Cardiothorac Surg..

[9] Gonzalez-Rivas D, Fernandez R, Fieira E, Rellan L (2013). Uniportal video-assisted thoracoscopic bronchial sleeve lobectomy: first report. J Thorac Cardiovasc Surg..

[10] Gonzalez-Rivas D, Delgado M, Fieira E, Pato O (2014). Left lower sleeve lobectomy by uniportal video-assisted thoracoscopic approach. Interact Cardiovasc Thorac Surg..

[11] Gonzalez-Rivas D, Fieira E, de la Torre M, Delgado M (2014). Bronchovascular right upper lobe reconstruction by uniportal video-assisted thoracoscopic surgery. J Thorac Dis..

[12] Huang J, Li J, Qiu Y, Xu X, Sekhniaidze D, Chen H, Gonzalez-Rivas D, He J (2015). Thoracoscopic double sleeve lobectomy in 13 patients: a series report from multi-centers. J Thorac Dis..

[13] Gonzalez-Rivas D, Delgado M, Fieira E, Fernandez R (2014). Double sleeve uniportal video-assisted thoracoscopic lobectomy for non-small cell lung cancer. Ann Cardiothorac Surg..

[14] Wang X, Jiao W, Zhao Y, Xuan Y, Wang Z (2015). Two-incision approach for video-assisted thoracoscopic sleeve lobectomy treating the central lung cancer. Indian J Cancer..

[15] Detterbeck FC, Boffa DJ, Tanoue LT (2009). The new lung cancer staging system. Chest..

[16] Walker WS, Codispoti M, Soon SY, Stamenkovic S, Carnochan F (2003). Long-term outcomes following VATS lobectomy for non-small cell bronchogenic carcinoma. Eur J Cardiothorac Surg..

[17] McKenna RJ, Houck W, Fuller CB (2006). Video-assisted thoracic surgery lobectomy: experience with 1,100 cases. Ann Thorac Surg..

[18] Boffa DJ, Allen MS, Grab JD, Gaissert HA, Harpole DH (2008). Data from The Society of Thoracic Surgeons General Thoracic Surgery database: the surgical management of primary lung tumors. J Thorac Cardiovasc Surg..

[19] Ettinger DS, Akerley W, Bepler G, Blum MG, Chang A (2010). Non-small cell lung cancer. J Natl Compr Canc Netw..

[20] Santambrogio L, Cioffi U, De Simone M, Rosso L, Ferrero S (2002). Video-assisted sleeve lobectomy for mucoepidermoid carcinoma of the left lower lobar bronchus: a case report. Chest..

[21] Kamiyoshihara M, Nagashima T, Igai H, Atsumi J, Ibe T (2011). Video-assisted thoracic lobectomy with bronchoplasty for lung cancer, with special reference to methodology. Interact Cardiovasc Thorac Surg..

[22] Li Y, Wang J (2013). Video-assisted thoracoscopic surgery sleeve lobectomy with bronchoplasty: an improved operative technique. Eur J Cardiothorac Surg..

[23] Fadel E, Yildizeli B, Chapelier AR, Dicenta I, Mussot S (2002). Sleeve lobectomy for bronchogenic cancers: factors affecting survival. Ann Thorac Surg..

[24] Yildizeli B, Fadel E, Mussot S, Fabre D, Chataigner O (2007). Morbidity, mortality, and long-term survival after sleeve lobectomy for non-small cell lung cancer. Eur J Cardiothorac Surg..

[25] Sugi K, Kaneda Y, Esato K (2000). Video-assisted thoracoscopic lobectomy achieves a satisfactory long-term prognosis in patients with clinical stage IA lung cancer. World J Surg..

[26] Watanabe A, Mishina T, Ohori S, Koyanagi T, Nakashima S (2008). Is video-assisted thoracoscopic surgery a feasible approach for clinical N0 and postoperatively pathological N2 non-small cell lung cancer?. Eur J Cardiothorac Surg..

[27] Scott WJ, Allen MS, Darling G, Meyer B, Decker PA (2010). Video-assisted thoracic surgery versus open lobectomy for lung cancer: a secondary analysis of data from the American College of Surgeons Oncology Group Z0030 randomized clinical trial. J Thorac Cardiovasc Surg..

[28] Kutlu CA, Goldstraw P (1999). Tracheobronchial sleeve resection with the use of a continuous anastomosis: results of one hundred consecutive cases. J Thorac Cardiovasc Surg..

[29] Tedder M, Anstadt MP, Tedder SD, Lowe JE (1992). Current morbidity, mortality, and survival after bronchoplastic procedures for malignancy. Ann Thorac Surg..

[30] Merritt RE, Mathisen DJ, Wain JC, Gaisser HA, Donahue D (2009). Long-term results of sleeve lobectomy in the management of non-small cell lung carcinoma and low-grade neoplasms. Ann Thorac Surg..

